# Improving safety in care homes: protocol for evaluation of the Walsall and Wolverhampton care home improvement programme

**DOI:** 10.1186/s12913-017-2013-x

**Published:** 2017-01-26

**Authors:** Sarah Damery, Sarah Flanagan, Kiran Rai, Gill Combes

**Affiliations:** Institute of Applied Health Research, College of Medical and Dental Sciences, University of Birmingham, Edgbaston, Birmingham, West Midlands B15 2TT USA

**Keywords:** Safety, Improvement, Care homes, Evaluation, Training, Mixed methods

## Abstract

**Background:**

Improving safety in care homes is becoming increasingly important. Care home residents typically have multiple physical and/or cognitive impairments, and adverse events like falls often lead to hospital attendance or admission. Developing a safety culture is associated with beneficial impacts on safety outcomes, but the complex needs of care home residents, coupled with staffing pressures in the sector, pose challenges for positive safety practices to become embedded at the individual and organisational levels. Staff training and education can positively enforce safety culture and reduce the incidence of harms, but improvement initiatives are often short lived and thorough evaluation is uncommon. This protocol outlines an evaluation of a large-scale care home improvement programme in the West Midlands.

**Methods:**

The programme will run in 35 care homes across Walsall and Wolverhampton over 24 months, and we anticipate that 30 care homes will participate in the evaluation (*n* = 1500 staff). The programme will train staff and managers in service improvement techniques, with the aim of strengthening safety culture and reducing adverse safety event rates. The evaluation will use a pre-post design with mixed methods. Quantitative data will focus on: care home manager and staff surveys administered at several time points and analysis of adverse event rates. Data on hospital activity by residents at participating care homes will be compared to matched controls. Qualitative data on experience of training and the application of learning to practice will be collected via semi-structured interviews with staff (*n* = 48 to 64) and programme facilitators (*n* = 6), and staff focus groups (*n* = 36 to 48 staff). The primary outcome measure is the change in mean score on the safety climate domain of the Safety Attitudes Questionnaire between baseline and programme end.

**Discussion:**

This mixed methods evaluation of a large-scale care home improvement programme will allow a substantial amount of qualitative and quantitative data to be collected. This will enable an assessment of the extent to which care home staff training can effectively improve safety culture, lower the incidence of adverse safety events such as falls and pressure ulcers, and potentially reduce care home resident’s use of acute services.

## Background

In recent years, an important goal for health services worldwide has been the development of a positive patient safety culture. Safety culture refers to the way that patient safety is considered within an organisation and the structures and processes put in place to support it [1]. It is believed that developing a positive safety culture in healthcare organisations is associated with a beneficial impact on safety outcomes and a lower incidence of clinical and other errors that may result in harm. A number of factors may contribute to a positive safety culture, including staffing levels; staff awareness of safety and training; staff willingness to improve safety; staff beliefs in their own ability to improve safety; systems for monitoring risk, and systems for reporting adverse events [2].

Most research into safety culture improvement has focused on hospitals, and the concept has only recently emerged in other areas of health and social care provision such as the care home sector [3]. This is surprising given the complex needs of care home residents who often have multiple physical, cognitive and sensory impairments [4]. A recent UK care home census reported that 87% of residents have high support needs, defined as having one or more of dementia, confusion, challenging behaviour, dual incontinence, severe hearing/visual impairment or dependence in mobility [5]. In this population, adverse safety events can quickly escalate and lead to hospital attendance or admission [6]. The care home sector is also characterised by frequent policy and regulatory changes, heavy workloads, high staff turnover and difficulty recruiting and retaining competent staff [3]. These factors make it challenging for positive safety practices to be cascaded to staff and to become embedded within care home organisational culture [6].

The nature of the care home resident population, coupled with workforce issues within the sector has meant that quality and safety in care homes is becoming an increasingly important concern for adult social care. The most common adverse safety events in care homes are accidental injuries involving residents and staff, pressure ulcers, falls, wounds and medication errors [7–9]. A limited number of safety and quality improvement initiatives have been developed and tested in the care home sector, showing some evidence of effectiveness. Improving falls awareness and training care home staff in falls reduction can significantly lower the incidence of falls [6]. Studies focusing on pressure ulcer prevention have shown that incidence is associated with staffing levels and staff education/knowledge, and that modest reductions can be achieved by raising staff awareness, implementing training and regularly monitoring risk [10, 11]. A further study reported a 62% reduction in pressure ulcer incidence when education was combined with a multidisciplinary approach to prevention and the introduction of systematic recording procedures [12]. Similarly, a large study in 39 US states found that quality improvement methods were associated with a significant reduction in grade 3–4 pressure ulcers [13].

However, quality and safety improvement approaches in care homes are often small-scale and improvements may not persist in the longer term. For this reason, it is often unclear how successful approaches can be incorporated into routine practice. This protocol outlines a mixed methods evaluation of a large-scale care home improvement programme being undertaken in the West Midlands which will provide training in service improvement techniques to care home staff and managers, with the aim of strengthening safety culture and reducing the incidence of adverse safety events.

## Methods and design

### The care home improvement programme

The programme being evaluated has been developed by the West Midlands Patient Safety Collaborative (PSC), and is being designed and delivered in collaboration with Walsall and Wolverhampton Clinical Commissioning Groups (CCGs). It will run in 35 care homes across Walsall and Wolverhampton (all of which provide both residential services and nursing care) over 24 months. There are two main elements: first, training events and workshops will help care home staff and managers develop relevant skills and enhance their understanding of safety-related service improvement. Training will explore clinical and human risk factors related to safety, alongside techniques for designing and implementing service improvements. Second, facilitated sessions delivered in participating care homes will support staff to implement changes to practice relating to specific safety concerns such as falls prevention and pressure ulcer management.

### Programme evaluation

**Fig. 1:**
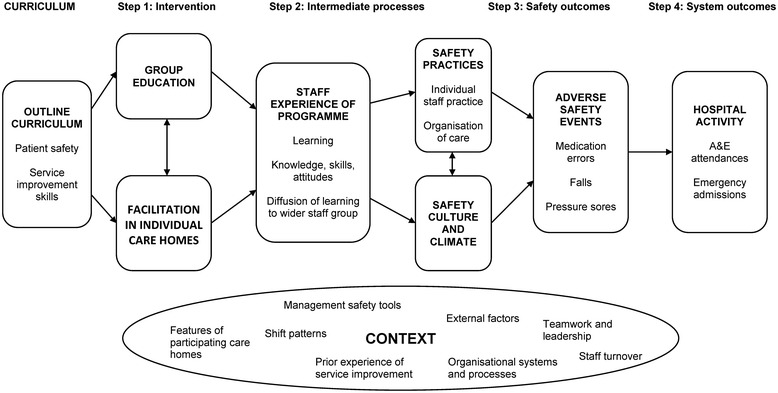
Programme theory

Service improvement initiatives are often complex and may be effective in part, or in certain circumstances only [14]. Initiatives designed to improve safety are challenging to evaluate because they typically use multi-faceted interventions and simple cause-effect relationships between interventions and outcomes are rarely evident. Consequently, the evaluation will take a multi-level approach [2] to include both formative and summative elements. The formative element will describe and analyse programme implementation and impacts on staff learning and changes to safety practices during the first 12 months. The summative element will combine multiple data sources at the end of the programme to: a) assess how well it achieved its objectives, b) identify the circumstances in which it was most/least successful, and c) assess the degree to which it was associated with changes in care home safety climate, adverse event rates and hospital activity. The evaluation design draws on Kirkpatrick’s framework for evaluating improvement initiatives that involve staff training [15] and follows a pre-specified programme theory which hypothesises the possible causal chain between the intervention and outcomes of interest (Fig. 1) [16].

### Aim and objectives

The overall aim is to assess the extent to which safety climate in care homes can be improved and the incidence of adverse events reduced, by skilling-up care home staff in service improvement techniques and providing facilitation to enable care homes to implement changes to practice. It will take a longitudinal approach, using a before and after study design. Specific objectives are to:

1. Describe how the programme is implemented over time and in multiple care homes
2. Assess staff experience of the programme
3. Identify what staff learn about safety and service improvement as a result of the programme
4. Identify changes that individual staff make to their practice
5. Assess facilitators and barriers to the application of staff learning to practice
6. Identify changes that staff teams and care homes make to safety-related processes
7. Analyse the impact on safety outcomes (safety climate, adverse events, hospital activity)
8. Identify any associations between the features of care homes and outcomes
9. Identify unintended consequences of the programme and assess how these may influence outcomes
10. Compare care homes which change the most with those that change the least, to identify the contexts and circumstances where the programme is more or less likely to be effective

### Data collection overview

The evaluation will use multiple data collection measures and draw on multiple datasets, as there may not be a direct relationship between the programme and adverse safety events in participating care homes. Indeed, absolute numbers of adverse events before and after the programme are likely to remain relatively low regardless of programme effectiveness. Adverse event rates may also rise rather than fall – staff training may facilitate increased event recording, or recording systems may themselves improve, not because the underlying incidence of adverse events increases [17]. To allow these relationships to be fully explored, evaluation data will be collected using both qualitative and quantitative methods.

### Outcome measures

#### Primary outcome measure

The primary outcome measure is the change in mean scores in the safety climate domain of the Safety Attitudes Questionnaire (SAQ) between baseline and the end of the programme. The SAQ was originally developed for use in the acute sector and has subsequently been validated for use in care homes [18, 19]. The tool has both long (60 item) and short (30 item) versions, but as benchmarking data have only been published for the 30 item version [20], the short form SAQ will be used. The SAQ measures six domains: teamwork climate, safety climate, job satisfaction, stress recognition, perception of management and working conditions, eliciting attitudes on a five point Likert scale. Responses are converted to a scale ranging from 0 to 100, and scores for each domain are calculated as the mean score of its component items, with higher scores denoting more positive safety attitudes [20]. The SAQ will be administered to managers and staff as part of larger surveys assessing experience of safety improvement initiatives at baseline, 12 and 24 months.

#### Secondary outcome measures

1. Adverse safety events: Changes over time in the incidence of falls, pressure ulcers, urinary tract infections (UTIs), infection control issues and medication errors will be measured, by analysing data that each care home routinely provides to their CCG. Analysis will assess annual rates of adverse safety events: a) for individual care homes, and b) across all care homes participating in the programme at 12 months prior to baseline (month 0), months 1–12, and months 13–24.
2. Hospital activity: Anecdotal evidence suggests that care home residents comprise around 30% of emergency hospital admissions in the West Midlands. Since it is plausible that safety improvements could reduce preventable hospital admissions, the evaluation will assess whether there are annual changes from baseline rates of A&E attendances and emergency admissions from care homes participating in the programme (12 months prior to baseline, months 1–12 and months 13–24). As routine hospital datasets do not distinguish between care home admissions and those from a private residence [21], data will be obtained from NHS Digital using each care home’s postcode as an indicator of attendance/admission from that location. As each care home will share its postcode with up to 21 nearby private residences [22], searches will be limited to patients aged 75 and over to attempt to distinguish hospital activity from care homes from activity by residents of surrounding properties. Hospital activity data will also be obtained for a matched control group of care homes not participating in the safety programme, to allow an overview of hospital activity trends independent of the programme. Matching will be carried out on a 1:1 basis with controls matched on care home size (number of beds), registration type, and Care Quality Commission (CQC) rating.

#### Intermediate outcomes

1. *Staff and facilitator experience*: Data on staff experience of the programme (learning, knowledge, attitudes and skills) will be collected using qualitative and quantitative methods. Qualitative data will include semi-structured interviews with programme facilitators and CCG managers at months 6, 12 and 24, and focus groups with care home staff at 6, 12 and 18 months. Quantitative data will be collected using care home staff questionnaires at 12 and 24 months, and via analysis of feedback sheets returned by staff attending programme training.
2. *Changes to safety practices (care home level):* Semi-structured interviews with care home staff in four case study care homes (two in each region) in months 12 and 24 will collect qualitative data. Quantitative data will be collected from care home managers via questionnaires (‘questionnaire 1a’ at baseline and ‘questionnaire 1b’ at 12 and 24 months). These will establish the characteristics and features of each care home and whether or not there have been any general service improvement initiatives or safety-specific initiatives undertaken in the care home in the previous 12 months.
3. *Changes to safety practices (staff level):* Qualitative data will be collected via focus groups at 6, 12 and 18 months with staff who have attended training sessions, and via semi-structured interviews (months 12 and 24) with staff in each of four case study care homes. Care home staff questionnaires (‘questionnaire 2a’ at baseline and ‘questionnaire 2b’ at 12 and 24 months), will collect quantitative data to explore changes to individual practice that may be attributed to the programme.

### Care home recruitment

#### Recruitment to the evaluation

The programme will be implemented in 35 care homes across Walsall and Wolverhampton. The manager and/or owner of each care home will be approached by the evaluation team to provide signed consent for their care home to participate in the evaluation. Each care home will also be asked to consent for the evaluation team to access data on adverse safety event rates that are routinely provided to the CCG. It is anticipated that around 30 care homes will agree to participate in the evaluation.

#### Recruitment of case study sites

Four care homes (two in Walsall, two in Wolverhampton) will be selected as case study sites [23]. Case studies will be selected to provide maximum variation across sites in terms of care home size, CQC rating, ownership (whether part of a large or small chain or a single care home), and specific care home features that may impact on safety such as resident profile. Once selected, the manager and/or owner of the potential case study sites will be approached to provide consent for their participation.

### Quantitative data collection

Routine datasets will be used wherever possible, to minimise the burden of care home participation in the evaluation and to maximise data reliability. Table 1 summarises the quantitative data to be collected, its purpose, and the time points for data collection.

**Table 1:** Quantitative data collection

Data type	Data tool	Data source	Detail of data to be collected	Time point(s)
Newly collected data	Questionnaire 1a	Care home managers (*n* = 30)	•Care home features (number of beds, registration type, staff by grade, hours and shift, shift patterns, GP support arrangements, CQC ratings) •Demographic information •Prior experience of care home safety and service improvement initiatives •Safety Attitudes Questionnaire	Baseline
	Questionnaire 1b	Care home managers (*n* = 30)	•Care home features (number of beds, registration type, staff by grade, hours and shift, shift patterns, GP support arrangements, CQC ratings) •Demographic information •Safety Attitudes Questionnaire •Experience of the programme, learning about safety, implementation of any service improvement or safety-related changes at the care home following the programme	12, 24 months
	Questionnaire 2a	All (non-manager) staff at participating care homes	•Demographic information •Safety Attitudes Questionnaire •Prior experience of safety and service improvement initiatives	Baseline
	Questionnaire 2b	All (non-manager) staff (regardless of attendance at training events or care home-based sessions)	•Demographic information •Safety Attitudes Questionnaire •Experience of the programme if the staff member participated; learning about safety and application of learning to individual practice	12, 24 months
	Session feedback sheets	All staff attending training and care home-based sessions	•Tick-box feedback sheets about the session attended	Each training or care home-based session
Routinely collected data	Adverse safety events	Data that care homes supply to CCG	•Rate of adverse safety events at each care home in the 12 months prior to each data collection point (pressure ulcers, falls, UTIs, infection control issues, medication errors, GP visits to each care home)	12 months before baseline, 12, 24 months
	Hospital activity	NHS Digital to provide via postcode searching of national datasets	•A&E attendance in the 12 months before each data collection point •Emergency admission rates in the 12 months before each data collection point	12 months before baseline, 12, 24 months

#### Sample size

The evaluation is powered to detect a 5-point change in mean score on the safety climate domain of the SAQ between baseline and end of study. Care home sign up to the evaluation is ongoing, so the study population is currently unknown. Research into typical care home staffing levels shows that 23% employ <11 staff, around 53% employ 11–49 staff, 13% employ 50–199, and <1% of care homes have more than 200 staff [24]. Taking this into account, the study is powered on the assumption that there will be 30 participating care homes, each with an average of 50 staff, giving a sampling frame of 1,500 individuals. Survey response rates in the care home sector are typically low [19]. Assuming a 25% response, and factoring in an expected 20% attrition rate (six care homes withdrawing between baseline and month 24), we would expect 375 survey responses at baseline and 300 at the end of the evaluation. Assuming a 20-point standard deviation in mean SAQ safety climate scores across respondents [20], this sample size would be sufficient to detect a 5-point change in mean SAQ safety climate score at 80% power and 5% significance (250 responses needed in each group, 500 overall). (Sample size calculated using EpiCalc 2000, version 1.02).

### Survey of care home managers (questionnaires 1a and 1b)

#### Sampling and recruitment

A survey pack will be delivered to the manager of each participating care home (*n* = 30). The pack will contain the survey (questionnaire 1a at baseline; questionnaire 1b at 12 and 24 months), a covering letter and a reply-paid envelope for survey return. Non-responders will receive a reminder pack 4 weeks after the initial approach.

#### Data collection

Questionnaire 1a will collect data on care home features and characteristics (size, registration, number of staff by grade and shift patterns, CQC ratings), demographic information (age, qualifications), prior experience of care home safety and service improvement initiatives, and the SAQ. Questionnaire 1b will include the SAQ and ask for the same care home and demographic information as questionnaire 1a but questions about prior experience of safety/service improvements will be replaced by questions about attendance at programme training events and whether any service or safety-related improvements were implemented following programme participation.

### Survey of (non-manager) care home staff (questionnaires 2a and 2b)

#### Sampling and recruitment

Each care home manager will be asked to provide the names of all employees. A survey pack addressed to each named individual will be delivered to participating care homes. The pack will contain the survey (questionnaire 2a at baseline and questionnaire 2b at 12 and 24 months), covering letter and reply-paid return envelope. Non-responders will receive a reminder pack 4 weeks after the initial approach.

#### Data collection

Questionnaire 2a will collect data on the SAQ, staff members’ prior experience of safety and service improvement initiatives and selected demographic information (role, age, qualifications, length of time working at the care home. Questionnaire 2b will be the same as questionnaire 1a, with questions on prior experience of safety or service improvements replaced by questions about programme training attendance and whether learning has been applied to individual practice.

### Training and care home-based session feedback sheets

At all training events and care home-based sessions, facilitators will hand out feedback sheets for completion by attendees, to collect data on reasons for attending the training, perceptions of training effectiveness, key learning points and feedback as to what could have been done better.

### Adverse safety events

All care homes in Walsall and Wolverhampton regularly provide routine data on adverse safety events to their respective CCG. The evaluation team will obtain routine data on pressure ulcers, falls, UTIs, infection control, medication errors and numbers of GP visits to each care home at baseline (covering the 12 months before the programme), at 12 months (covering year one), and 24 months (year two).

### Hospital activity

The procedure for collecting data on care home residents’ hospital activity is described under secondary outcome measures.

### Qualitative data collection

Table 2 summarises the qualitative data collection.

**Table 2:** Qualitative data collection

Type of data	Data source	Detail of data to be collected	Time point(s)
Non-participant observation of training sessions	Observation, training sessions	•Observation of the training and care home-based sessions will allow recording of content and delivery of training, length of sessions, number of attendees. Also, identification of possible issues for follow-up in semi-structured interviews in the case study care homes	Each session
Non-participant observation of care home-based sessions	Observation, care home-based sessions		A selected number of sessions throughout the programme (approximately 30% of the total)
Documentary analysis	Patient Safety Collaborative Programme Board	•All documents relating to the programme will be analysed to provide information about programme planning, content and delivery	Throughout the programme
Focus groups	Care home staff participating in the programme (2 groups of 6–8 staff at each time point)	•What has been learned by participation in the programme, how learning has been applied to individuals’ practice, barriers and facilitators to implementing change in the care home, perceptions of best/worst features of the programme, suggestions for improvement	6, 12 and 18 months
Semi-structured interviews	CCG managers and programme facilitators (*n* = 6)	•Experience of running the programme, perceived barriers to safety-related change in care homes, best/worst features of the programme, suggestions for improvement	Months 6, 12 and 24
Semi-structured interviews in four case study care homes	Staff and care home managers (participants and non-participants in the programme) (*n* = 6 to 8 in each of the four case study sites at each time point)	•What has been learned, how learning has been applied to individuals’ practice, barriers and facilitators to implementing change in the care home, changes made to safety-related processes at the care home level, extent and type of collaboration across care homes	12 and 24 months

### Non-participant observation (training events and care home-based sessions)

All training events and approximately 30% of care home-based sessions (selected to cover a range of care home sizes, registration types and CQC ratings) will be observed by members of the evaluation team. Observations will allow a detailed description of the content and delivery of the programme (e.g. the length of sessions, issues covered, number of attendees), as well as identifying possible issues for follow-up in semi-structured interviews at each case study site.

### Documentary analysis

All documents relating to the programme will be obtained from the PSC Programme Board and analysed to provide a detailed description of the motivation behind the programme, its design, and objectives. Analysis of programme documents will also allow the programme theory to be refined.

### Focus groups

#### Sampling and recruitment

Staff who have participated in training events and/or care home-based sessions will be eligible to take part in a focus group of between 6 to 8 staff. Staff will be identified via sign-up sheets provided at each session by programme facilitators. The evaluation team will post a focus group recruitment pack to selected staff – ensuring a range of staff roles and care home characteristics are represented in the sample. The recruitment pack will include a participant information sheet (PIS), covering letter, reply slip and reply-paid envelope for reply slip return.

#### Data collection

Six focus groups will be run: two at 6 months, two at 12 months, and two at 18 months into the programme. Focus groups will explore staff experience of the programme including what has been learned and how learning has been applied to practice, perceptions of the barriers/facilitators of effecting safety-related change in their care home, and suggestions for programme improvement. Each focus group will be audio-recorded and will last approximately 90 min. Recordings will be transcribed verbatim and transcripts proof-read against the recordings by one of the evaluation team members who facilitated the group. To ensure that staff are not unduly burdened, different staff members will be recruited to the focus groups at each of the three study time points.

### Semi-structured interviews with CCG managers and programme facilitators

CCG managers and programme facilitators (*n* = 6) will be invited to attend a semi-structured interview at months 6, 12 and 24. Interviews will explore participant’s experience of running the programme, perceived barriers to safety-related change in care homes, perceptions of the best and worst features of the programme and suggestions for programme improvement. Interviews will take place face-to-face or over the telephone and will last for approximately 20–30 min. Interviews will be audio-recorded, and recordings will be transcribed verbatim. Transcripts will be proof-read against recordings by the member of the evaluation team who conducted the interview.

### Semi-structured interviews with staff and care home managers in case study sites

#### Staff recruitment

Semi-structured interviews with staff employed at each case study care home will be undertaken in months 12 and 24 (6 to 8 interviews at each case study site at each time point; *n* = 48 to 64 total). Interview packs including a PIS, reply slip and reply-paid envelope will be delivered to staff at each case study care home. Of the staff willing to be interviewed, a range of individuals occupying different job roles/grades and those working evening/night/weekend shifts will be selected to ensure a diverse sample. Training programme participation is not a pre-requisite for eligibility. Interviews will take place either face-to-face at the care home or over the telephone. To take account of staff turnover, staff will be resampled for interview at 24 months, to ensure that the views of more recently recruited staff are included.

#### Data collection

The topic guide will cover:

- Methods that staff have used to prioritise and implement safety improvements in their workplaces
- Whether safety-related activities and/or other service improvements have been undertaken, why and by whom
- Which improvements have been easiest and most difficult to implement, and why
- How skilled and confident staff have felt in using service improvement techniques
- What activities, if any, have been stopped as a result of the programme
- Whether staff have shared information with employees from other care homes in the programme

Interviews will last for 20–30 min and will be audio-recorded. Recordings will be transcribed verbatim and transcripts read against recordings by the evaluation team member who conducted the interview.

### Data analysis

#### Quantitative data

Questionnaire analysis will be descriptive and will compare numbers and percentages of survey responses across the different data collection time points. Data from questionnaires 1a and 1b will be analysed against adverse event rates obtained from the CCG data using comparison of proportions tests to assess whether specific adverse events are associated with particular care home features (e.g. size, staffing, CQC rating).

Data from staff questionnaires will be used alongside training session feedback sheets and qualitative data to assess staff learning and changes to safety-related practices made as a result of the programme. Mean changes in SAQ scores over time will be assessed: a) for each care home, b) for the entire group of participating care homes, c) according to key characteristics of participating care homes (including CCG area), and d) by staff job role. Sub-group analyses will use t-tests to compare SAQ safety climate domain scores between groups, with post-hoc tests undertaken as appropriate to determine where differences between specific sub-groups lie. If the sample size permits predictive modelling of SAQ scores according to care home features and staffing factors, care homes will be accounted for in the analysis as fixed effects to control for clustering.

Descriptive analysis of changes in adverse event rates will be undertaken: for individual event types (falls, pressure ulcers etc.) and for total numbers of events, alongside an analysis of changes over time in A&E attendances and emergency hospital admission rates for the year pre-dating the programme and in the two years following programme implementation. Hospital activity data will be compared to the matched control care homes to assess whether trends observed in participating care homes are occurring independently of the wider context and thus may be attributable to the safety programme.

#### Qualitative data

Data from interviews and focus groups will be analysed thematically [25]. At least two members of the team will analyse and independently code at least 10% of the interview/focus group transcripts, with results compared and discussed until agreement is reached. The resulting codes and themes will be refined and elaborated as more data are collected. Where data does not fit existing themes, new themes will be developed or existing ones modified until all data can be coded.

#### Data synthesis

Although the quantitative and qualitative data will initially be analysed separately, queries generated by these analyses will lead to further analysis and the synthesis of findings derived via different methodologies. Triangulation across multiple datasets [26] will enable rich descriptions of the case studies to be developed and analysis will relate emerging findings back to the programme theory.

## Discussion

Improving safety in the care home setting is a key priority for the adult social care sector. Care home residents represent a population with multiple physical and cognitive impairments who may be particularly vulnerable to – often preventable - adverse events such as falls, pressure ulcers, UTIs and medication errors. Although the importance of care home resident safety is increasingly recognised, few initiatives to improve staff knowledge, skills and awareness have been implemented in the care home setting and thorough evaluation is uncommon. The few safety improvement initiatives that have been attempted have often been limited in scale and scope, and the longevity of any observed safety and quality improvements is frequently unknown, as are the specific elements of care home interventions that may be more or less effective. This evaluation of the Walsall and Wolverhampton Care Home Improvement Programme provides an opportunity to assess a large-scale safety improvement programme in detail over a two-year period. By using a multi-level, mixed methods approach, the evaluation will allow a large amount of qualitative and quantitative data to be collected, enabling an assessment of the extent to which care home staff training can effectively improve safety culture, lower the incidence of adverse safety events, and potentially reduce care home residents’ use of acute sector services.

## Abbreviations

A&E: Accident and emergency
CCG: Clinical commissioning group
CQC: Care quality commission
GP: General practitioner
NHS: National health service
PSC: Patient safety collaborative
SAQ: Safety attitudes questionnaire
UK: United Kingdom
US: United States
UTI: Urinary tract infection
